# Integrated multi-omics and genome mining strategies for activating cryptic polyketide biosynthetic gene clusters in *Streptomyces*: an overview

**DOI:** 10.3389/fmicb.2026.1944089

**Published:** 2026-09-30

**Authors:** R. Santhoshkumar, Hafid Mohamed, Martin Jemo

**Affiliations:** 1 College of Agriculture and Environmental Science (CAES), University Mohammed VI Polytechnique (UM6P), Benguerir, Morocco; 2 Faculté des Sciences Semlalia, Laboratoire d'Excellence en Agrobiotechnologie et Bioingénierie, Centre AgroBiotech, Unité de Recherche Labellisée CNRST (URL05-CNRST), Université Cadi Ayyad, Marrakech, Morocco

**Keywords:** artificial intelligence (AI), base editing, CRISPR, heterologous expression, multi-omics, polyketides, Streptomyces

## Abstract

Beneficial microbes from soil or marine habitats are promising sources for discovery of new bioactive compounds for medicine and agriculture, largely through biosynthetic gene clusters (BGCs) that encode the production of complex natural products for drug and antibiotic discovery. However, most BGCs are transcriptionally silent under standard testing conditions and may require a complex, integrated approach for activation. In this review, we revisit previous approaches to activating BGCs, focusing on polyketides. We additionally discuss how integrating genome mining strategies with multi-omics integration, synthetic biology methods, and precise genome-editing tools is providing increasingly precise strategies for the discovery and engineering of polyketide-producing *Streptomyces*. We further discuss how to couple artificial intelligence (AI)-designed promoters could enable the production of novel compounds with improved yields, expanded chemical diversity, and enhanced industrial potential. We further examine how state-of-the-art CRISPR-Cas systems, including base editing, facilitate precise editing of high-GC-content genomes while circumventing the genome instability and Cas9-associated cellular toxicity that frequently accompany double-strand break formation in *Streptomyces*, and how chromatin topology rewiring dismantles transcriptional silencing. Unlike reviews that address genome mining, pathway engineering, or genome editing as separate approaches, this review presents an integrated framework linking computational BGC prediction, multi-omics validation, and precision engineering to prioritize and activate cryptic polyketide pathways in *Streptomyces*. We propose comprehensive computational genomics prediction approaches and integrate them with experimental validation through promoter engineering, transcription factor decoys, and chassis strain optimization, highlighting how these methods and tools are advancing the field from descriptive BGC catalogs to scalable, predictive models for next-generation drug discovery. Future efforts to couple AI-generated, sequence-orthogonal promoter libraries with biology-based modeling hold great promise.

## Graphical abstract

**Figure:**
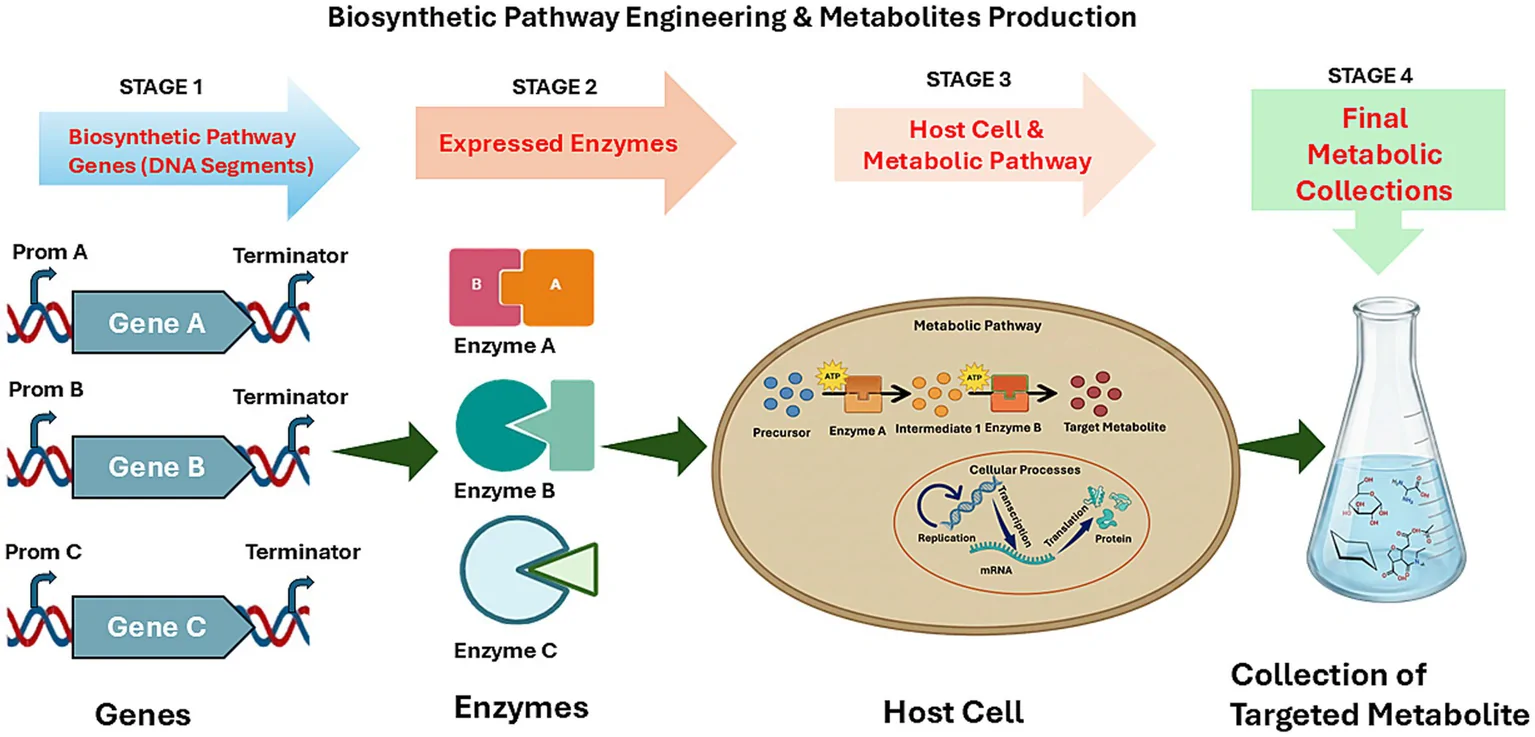

## 1 Introduction

Global health and sustainable agriculture depend broadly on microbial natural products, and more than 70% of antibiotics used in medicine are derived from natural sources, predominantly from *Streptomyces* (Krysenko, 2025; Sivalingam et al., 2024). Endophytic *Streptomyces* species are among the most promising sources of new metabolites. They live inside plants without harming them, and their biosynthetic repertoire reflects the unique ecological niches they occupy (Veilumuthu and Godwin, 2022). These bacteria synthesize various bioactive secondary metabolites, notably aromatic polyketides (a large and diverse group produced by complex biosynthesis with medicinal, agricultural, and industrial applications, including type II and III polyketides). This group comprises chemically diverse natural products, many of which are among the most clinically and industrially significant molecules globally (Yang et al., 2023). Generally, their biosynthesis involves short-chain acyl-CoA precursors (e.g., acetyl-CoA, malonyl-CoA) that undergo a carbon–carbon condensation to yield carbon frameworks bearing β-keto groups. Further genome mining in *Streptomyces* uncovers several biosynthetic gene clusters (BGCs), with 23–83 BGCs per genome (an average of approximately 44) reported across 121 genomes (Caicedo-Montoya et al., 2021). However, most BGCs remain unexpressed under conventional laboratory conditions (Liu et al., 2025). The remainder, generally referred to as “silent” or “cryptic” clusters, form a large yet unexploited source of chemical diversity in natural products (Covington et al., 2021). This difference reflects a critical biotechnological loss, as these clusters typically require specific natural triggers absent in standard laboratory cultures (Jeong et al., 2025; Alam et al., 2022).

Cryptic BGCs are biosynthetic gene clusters reported in microbial genomes that are typically transcriptionally silent or weakly expressed under standard laboratory conditions (Shil et al., 2026). Silent BGCs are a subset of cryptic BGCs in which low or absent expression results in the production of a very small amount of the corresponding natural product that cannot be detected (Covington et al., 2021). The term ‘orphan’ indicates that the genome sequence of the biosynthetic gene cluster has not been experimentally linked to a natural product (NP). While some BGCs are constitutively expressed and readily identified by their NP product under standard culture conditions, others may be difficult to detect (Covington et al., 2021). An unexpressed BGC is typically defined as a cluster that does not produce detectable mRNA transcripts under a specified set of growth conditions. BGCs are functional, genetically encoded pathways that often remain silent or unexpressed, so screening and activating them can unlock their potential to yield new natural products (Hoskisson and Seipke, 2020; Covington et al., 2021). Advances in genomic DNA sequencing have led to the discovery of thousands of new polyketides gene clusters, and experimentally characterizing and validating these newly identified pathways with respect to their biological activities poses significant challenges (Vandova et al., 2020). For example, *Streptomyces* species encode a highly conserved biosynthetic megacluster that produces four structurally distinct natural products, including stravidins, acidomycin, dapamycins, and 2-methyl-7-keto-8-aminopelargonic acid (α-Me-KAPA), along with the biotin-binding protein streptavidin (Gordzevich et al., 2026). Advances in genome sequencing, bioinformatics, and synthetic biology now provide unprecedented opportunities to unlock this hidden chemical potential (Groff-Vindman et al., 2025). For instance, integrative ‘genome-to-function’ pipelines now routinely couple whole-genome sequencing with cheminformatics and metabolomics to prioritize and validate cryptic BGCs before applying synthetic biology tools to overexpress the metabolites (Pattapulavar et al., 2026).

Polyketides are a large, diverse group produced by complex biosynthesis and have medicinal, agricultural, and industrial applications, including type II and III. In general, their synthesis involves polyketide synthases (PKSs), the core multidomain enzymes that carry out these central transformations. Subsequent tailoring enzymes, through their repertoire of modifications, including reduction, cyclization, aromatization, oxygenation, methylation, and glycosylation, allow for apparently unlimited structural and functional diversification (Yang et al., 2023). Recent advancements in synthetic biology have spurred efforts to discover novel metabolites. High-throughput sequencing, multi-omics integration, and precision genome editing now provide the tools to activate cryptic biosynthetic genes in *Streptomyces* to create predictable polyketide producers. Unlike recent reviews that have treated genome mining (Jeong et al., 2025; Alam et al., 2022), promoter engineering (Shil et al., 2026), and editing toolkits (Krysenko, 2025) as separate topics, this review integrates computational prediction, multi-omics validation, precision editing, activation triggers, and chassis optimization into a single end-to-end decision pipeline (Figure 1), and critically incorporates the most recent literature (2020–2026), defining an evidence hierarchy for BGC–metabolite linkage while addressing key translational bottlenecks. Additionally, we present an integrated approach that connects genomic prediction to experimental validation, advancing the field from descriptive observation to experimental models for next-generation natural product discovery. Therefore, the overall objective of this review is to examine how genomic predictions can be integrated with experimental validations that move beyond cataloging of BGCs, thus accelerating their use in drug discovery. The specific objectives were: (1) Assess existing activation approaches targeting polyketides. (2) Examine the role of advanced Evaluate how advanced CRISPR systems and nucleoid-level engineering strategies overcome the transcriptional silencing of cryptic BGCs. (3) Integrate computational predictions and experimental chassis optimizations in an end-to-end process pipeline. (4) Define an evidence hierarchy for BGC metabolite linkages to overcome translation bottlenecks.

**Figure 1:**
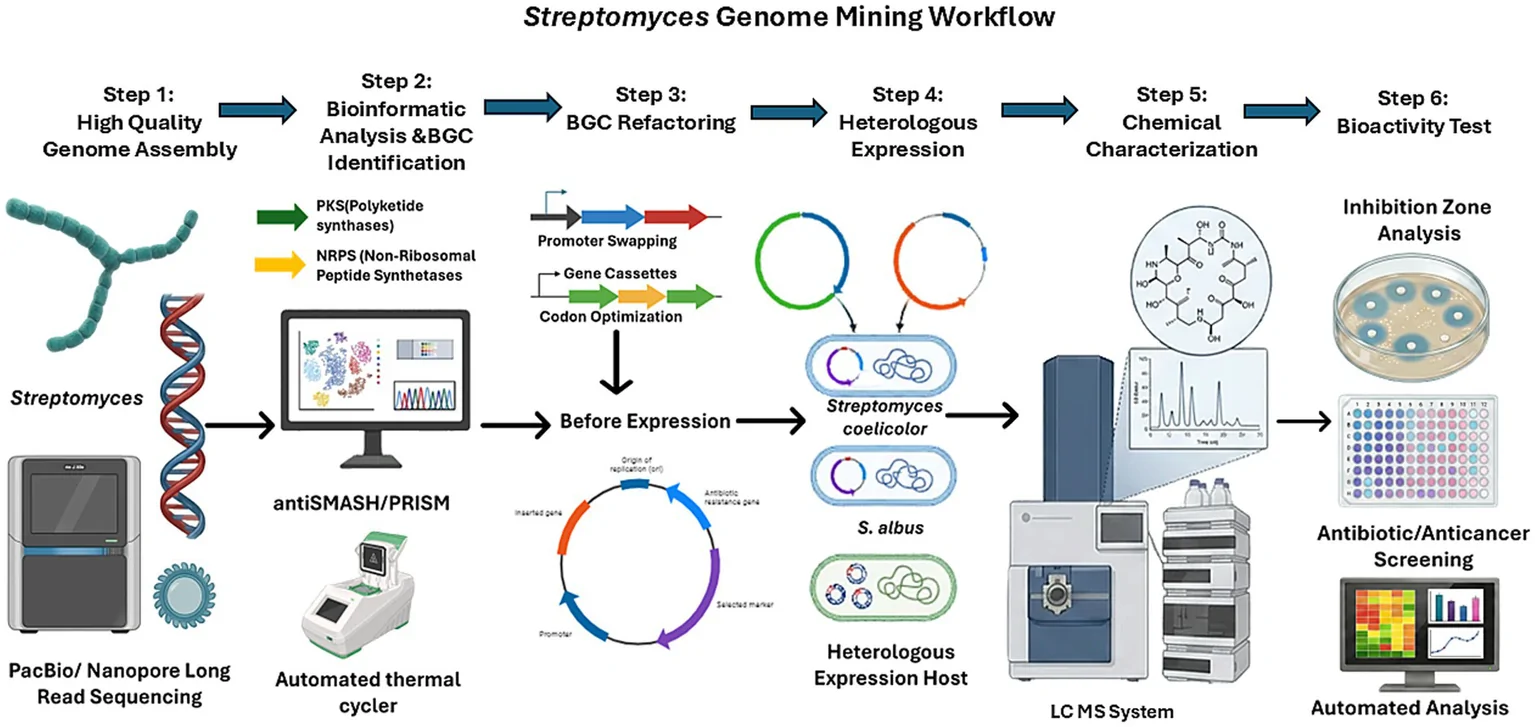
Schematic workflow for cryptic BGC discovery in *Streptomyces*. Step 1: High-quality genome assembly using PacBio/Nanopore long-read sequencing. Step 2: Bioinformatics BGC identification and classification of PKS and NRPS clusters using antiSMASH/PRISM. Step 3: BGC refactoring by promoter swapping, gene-cassette assembly, and codon optimization. Step 4: Heterologous expression in *Streptomyces coelicolor* and *S. albus*. Step 5: Chemical characterization of metabolites by LC-MS. Step 6: Bioactivity assessment by inhibition-zone assays and antibiotic/anticancer screening with automated analysis.

## 2 Approach

We retrieved studies published from 2020 to 2026 from the Web of Science (the United States),^1^ Google Scholar (United States),^2^ and Scopus (Netherland)^3^ using keyword combinations of “*Streptomyces*,” OR “genome mining,” OR “CRISPR,” OR “multi-omics,” OR “heterologous expression,” OR “polyketide,” OR “Biosynthetic Gene Clusters”. Peer-reviewed articles, reviews, and preprints describing novel platforms or genomes were included to capture rapid methodological advances, with preprints explicitly flagged in the reference list. We excluded conference abstracts without full text.

## 3 Genomic diversity: the vast, untapped cryptic potential of *Streptomyces* BGCs

The development of high-throughput sequencing techniques provides a powerful method for characterizing actinobacterial genomes. High-quality genome sequencing, combined with a systematic annotation approach, offers an exceptional prospect to investigate actinobacteria with extensive GC-rich genomes, which frequently encode complex metabolic systems that can be challenging to analyze without thorough genomic editing (Kumar and Dharumadurai, 2026).

Recent genomic surveys reveal that *Streptomyces* strains consistently harbor a vast diversity of biosynthetic gene clusters; for example, *Streptomyces* sp. IICT-RSP475 and *S. rubrogriseus* RKDTS3 encoded 18 and 21 BGCs responsible for producing ribosomally synthesized and post-translationally modified peptides (RiPPs), polyketides (PKS), non-ribosomal peptides (NRPS), and other secondary metabolites (Batchu et al., 2025; Kumar and Dharumadurai, 2026). This immense chemical diversity is further demonstrated by strains *S. albidoflavus* VIP-1, *S. maremycinicus*, and *Streptomyces* sp. AFD10 contains a larger number of BGCs, up to 35, dedicated to diverse structural antibiotics (Sedeek et al., 2025; Komaki et al., 2025; Fatima et al., 2024). These deep genomic analyses have always been dependent on simple counts of algorithmic clusters, which often, and mostly, overestimate actual biosynthetic capabilities. Most high-throughput workflows automatically identify near-functional or evolutionarily redundant distant BGCs; thus, a large unknown fraction of these “cryptic” loci might lack the essential architectural features for complete biosynthesis. The largest *Streptomyces* pangenome analysis to date encompassing 205 complete genomes also showed that although PKS and NRPS core enzymes were mostly strain-specific, primary metabolism pathways such as ectoine and hopanoids remained highly conserved (Otani et al., 2022).

The deep intra-species diversity is further highlighted by strain-level variation and hidden redundancy. For instance, *S. rapamycinicus* NRRL 5491 harbors 17 BGCs, notably including a duplicate polyketide cluster for rapamycin production (Jo et al., 2022), while *S. anandii* J6 contains multiple type II and III PKS clusters that remain functionally uncharacterized, paving the way for targeted CRISPR-Cas9 functional analysis (Kim and Kwak, 2025). Large-scale phylogenetic analyses of over 1,100 *Streptomyces* genomes confirm that BGC distribution is highly scattered and strain-dependent rather than species-conserved (Belknap et al., 2020). These studies highlight a critical challenge: traditional isolation methods capture only a fraction of this potential, forcing computational and multi-omics approaches to prioritize novel PKS activities.

## 4 Computational frameworks: machine learning for BGC prediction

Synthetic biology, a fast-growing field for natural product discovery over the past 20 years, has relied heavily on databases and computational tools to guide omics and genome-editing approaches (Bao et al., 2021). Sophisticated database platforms and tools have been developed and used to identify, annotate, and analyze BGCs directly from raw genome sequences. The antiSMASH v7.0 is the leading tool for detecting secondary metabolite biosynthetic gene clusters (SMGCs) in *Streptomyces* (Passari et al., 2025), using hidden Markov models and rule-based annotations to identify known and unknown BGCs (Blin et al., 2023).

However, traditional rule-based programs have mainly been used for determining established metabolic pathways. Recently, machine learning-powered tools like SanntiS, DeepBGC, and GECCO have been implemented for high-throughput genome mining to discover non-canonical and highly divergent BGCs (Sanchez et al., 2023; Carroll et al., 2021). Recently, several advances in deep learning models have been utilized to predict complex polyketide structures based on sequences. For example, an integration of extra-tree AT domain specificity prediction modeling approach used by Seq2PKS to accurately reconstruct the non-collinear Type I PKS pathways (Yan et al., 2024). Another recent development has been the application of transformer-based frameworks that treat protein domains as linguistic units toward unsupervised BGC prediction and the rational design of artificial clusters without predefined labels (Kawano et al., 2026). These models complement antiSMASH, which remains valuable for BGC-family prediction and classification. Similarly, GECCO and SanntiS can differentiate biosynthetic niches beyond existing knowledge with multidimensional, data-empowered BGC prediction. Other databases, MIBiG and the Secondary Metabolism Collaboratory (SMC), function as repositories for biosynthetic gene cluster (BGC) sequences and their associated annotations, aiding potential natural product discovery (Udwary et al., 2025).

StreptomeDB 4.0 is the most advanced database, containing over 8,500 natural products from approximately 3,900 *Streptomyces* strains and integrating antiSMASH-based BGC predictions with visual tools for NMR and MS spectra. The database includes over 336,000 natural product-protein associations and 400,000 predicted natural product-protein interactions, effectively aiding dereplication and advancing the discovery of new bioactive chemical species (Feng et al., 2025). Complementary bioinformatics platforms, such as PRISM (PRediction Informatics for Secondary Metabolomes) and BAGEL4, accelerate the systematic identification of BGCs and compare them with known reference clusters. Application of these tools to the *Streptomyces* strain CS-7 identified 36 BGCs, 25 of which corresponded to previously described clusters, indicating CS-7’s potential to produce novel metabolites (Alam et al., 2022).

Additionally, NegMDF, a workflow that combines mass defect filtering and bioinformatics, effectively closes the gap between predicted BGCs and detected metabolites. Applied to *S. cattleya* NRRL 8057, NegMDF rapidly identified 22 polyketides, including tetronate-containing cattleyatetronates (Liu et al., 2025). Mapping the coverage of these tools together enables the conversion of natural product workflows, which are traditionally sequences of individual experiments, into high-throughput parallel workflows (Table 1). Machine learning models tend to be biased toward familiar BGC families, which reduces their capability to recognize completely new, non-canonical chemical scaffolds (Lu et al., 2026). These computational advances and in silico predictions continue to have false-positive rates and cannot precisely determine the chemical structure of the final metabolite from the sequence alone.

**Table 1:** Computational tools and databases for BGC prediction and annotation in *Streptomyces*

Category	Name of tool/database	Function	Mechanism	Core elements	Original ref
Prediction tools	antiSMASH 7.0	Identification and classification of BGCs	Rule-based and HMMs	Rapid family determination, comparative analysis	Blin et al. (2023)
	DeepBGC	BGC discovery	Machine Learning	Detects novel clusters outside known logic	Hannigan et al. (2019)
	PRISM4	Identification and chemical prediction	Bioinformatics	Predicts chemical structures from BGCs	Skinnider et al. (2020)
	BAGEL4	Identification of RiPPs and bacteriocins	Bioinformatics	Specialized RiPP identification and characterization	van Heel et al. (2018)
	ARTS 2.0	Prioritization of BGCs via self-resistance gene detection	Bioinformatics/Comparative genomics	Identifies co-localized resistance genes within BGCs to flag bioactive clusters	Mungan et al. (2020)
Databases	MIBiG 3.0	Repository for BGC sequences	Curated database	Standardized BGC and annotation repository	Terlouw et al. (2023)
	SMC	BGC data sharing and analysis	Database	Community-driven BGC data sharing	Udwary et al. (2025)
	StreptomeDB 4.0	NP database with interactive visualization	Database	Integrates Streptomyces natural products with protein interactions and MS spectral information	Feng et al. (2025)
Genomic–metabolomic integration	NegMDF	Mass defect filtering + bioinformatics	Hybrid (Informatics + MS)	Connects genomic BGC predictions with negative-ion MS data for targeted polyketide discovery	Liu et al. (2025)

## 5 Multiomics integration: bridging the genotype-to-phenotype gap

High-throughput sequencing and multi-omics integration technologies have fundamentally transformed genome mining pipelines, establishing a new “deep mining” framework for linking genotypes to phenotypes (Wang et al., 2025).

### 5.1 Transcriptome-based metabolite gene signatures

Metabolic bottlenecks and regulatory switches in secondary metabolism can be efficiently detected by transcriptomic profiling. For example, comparative RNA-seq has been applied to show that the downregulation of specific polyketide synthase modules and certain central carbon metabolism pathways directly limits the supply of precursors such as malonyl-CoA, which, in turn, restricts the biosynthesis of polyketides (Huang et al., 2025; Liao et al., 2022). Besides, transcriptomics might indicate strategies for environmental activation; research shows that stressors such as ethanol shock can induce global metabolic reprogramming, leading to greater acetyl-CoA availability and the unlocking of cryptic biosynthetic clusters (Sekurova et al., 2022). Along with these transcriptional changes, it is possible to guide the rational design of interventions, such as overexpressing acetyl-CoA carboxylase, to restore reduced precursor pools and, in many cases, increase polyketide production.

### 5.2 Metabolomic and proteomic validation

Distinct culture conditions were systematically applied to stimulate metabolite production in *Streptomyces*, followed by LC–MS-based metabolomic analyses, which facilitated the identification and structural annotation of the compounds and their correlation with the corresponding BGCs (Tocino-Márquez et al., 2026). Untargeted metabolomics profiling showed that genome-edited *S. bambergiensis* AC-800 produced known and putatively novel compounds (Guerrero Garzón et al., 2025). Related studies catalogued secondary metabolites, including siderophores and macrolides (Kum and İnce, 2024; Sunghanghwa et al., 2025). However, a major methodological barrier remains: MS/MS features cannot be assigned confidently to cryptic BGCs without additional genetic validation.

Concurrently, label-free quantitative proteomics has been used to develop “proteomining” methods to identify active BGCs by correlating changes in protein abundance with secondary metabolite production (Gubbens et al., 2014). Proteomining identified BGCs responsible for calcium-dependent antibiotics, actinorhodin, undecylprodigiosin, and coelimycin P1 in *S. coelicolor* A3(2) (Linardi et al., 2022). The same approach also rediscovered the type II PKS responsible for albofungin production, a pathway previously identified through genome mining and heterologous-expression experiments (Linardi et al., 2022). At the translational stage, StrProQC serves as an approach to protein quality control, adapted to *Streptomyces* to facilitate translation of full-length messenger RNAs (mRNAs) into fully functional proteins, predominantly from larger genes such as Type I PKSs. The system employs a switch RNA and a cis-trigger riboregulator to ensure that translation is initiated once the ribosome detects a full-length distributed mRNA, enhancing the production of complex natural products such as spinosad and rapamycin up to 4.7-fold in heterologous hosts (Jiang et al., 2025).

Integrating genomics and metabolomics pipelines appears promising for elucidating secondary metabolomes; this approach was recently reported to reveal the activity of cryptic BGCs through LC–MS and GC–MS profiling. The complex MS/MS fragmentation profiles were correlated with algorithmic predictions of terpene and polyketide scaffolds to build a multi-omics strategy that could bridge the genotype (genome mining) and phenotype gap (metabolic profiling) (Pattapulavar et al., 2026). To further leverage these unique biosynthetic signatures, advanced “metabologenomic” targeting workflows that logically prioritize particular chemical scaffolds have recently emerged, including the successful discovery of novel β-branched conjugated polyketides displaying both anti-tubercular and anticancer activities through the combination of genomic hallmarks of HCS-homolog β-branching cassettes with metabolomic UV vibronic fine structure screening (Cui et al., 2026).

### 5.3 Evidence hierarchy for BGC–metabolite assignment

Multi-omics techniques generate hypotheses between BGCs and metabolites. However, transcriptome co-expression, MS-based annotation, correlation, or molecular networking do not prove that a single BGC produces a metabolite. Accordingly, assess BGC-metabolite assignments through a graded evidence hierarchy, from correlative evidence to genetic validation, heterologous expression, and structural confirmation.

*Correlative evidence:* BGC-metabolite pair candidates are identified via transcriptomics (RNA-seq), untargeted metabolomics (LC–MS/MS), and molecular networking (e.g., GNPS SIRIUS) based on co-occurrence or fragmentation correlation similarities. For example, molecular network analysis of *S. griseochromogenes* grown in three different systems discovered specific compounds in one of the growth systems. However, the appropriate metabolic pathways could not be discovered without additional analyses (Lindig et al., 2026). In a similar setup, the NegMDF approach was used to find 22 polyketides in the *S. cattleya* NRRL 8057 strain using mass spectrometric data, with BGCs and high-quality structural confirmation (Liu et al., 2025).

*Genetic deletion:* Targeted disruption of core biosynthetic genes, followed by loss of the corresponding metabolite, provides direct, fundamental evidence. Deleting kelB, kelC, and kelD from the competitive kel BGC in *S. hygroscopicus* SIPI-054 eliminated the pigment and increased milbemycin A3/A4 production by approximately 20% (Xu et al., 2026). Inactivating ∆accJ in *Streptomyces* sp. MA37 increased Accramycins A production by 330-fold (66 mg/L) and activated 11 accramycins A-K 1–11, naphthacemycin B1-12, and fasamycin C (Maglangit et al., 2020).

*Complementation (the restoration of function):* The reintroduction of deleted gene(s) followed by reinitiation of metabolite production provides compelling genetic proof that the phenotype is caused by the targeted gene rather than polar or off-target effects. Xu et al. (2026) confirmed that deleting the transABC operon inside the milbemycin BGC significantly reduced antibiotic production and biomass, whereas complementation restored both parameters to wild-type levels.

*Heterologous expression (improvement-of-function):* The expression of a novel BGC from a genetically distinct organism and synthesis of the target molecule offers independent evidence that the cluster is adequate for its production. Studies support this machinery in engineered *Streptomyces* hosts. Heterologous expression of a 211-kb BAC encompassing the concanamycin BGC in *S. avermitilis* SUKA32 resulted in the production of concanamycin and other aromatic polyketides, including the novel compound JBIR-157, demonstrating the host’s utility for accessing cryptic or near-silent loci (Kudo et al., 2024). The incorporation of a spi type II PKS BGC from marine *Streptomyces* spp. HDN155000 into *S. albus* J1074 via heterologous gene expression led to the synthesis of eight angucycline-related compounds, four of which are novel, and their structures were elucidated through chemical analyses utilizing tandem mass spectrometry and nuclear magnetic resonance (Zhang et al., 2024).

*Structural confirmation:* Structural characterization of metabolites using several complementary approaches, such as NMR and mass spectrometry, produces highly reliable data for confirming chemical structures. For example, Stictamycin was structurally elucidated and linked to an atypical type II PKS BGC through cloning and knockdown experiments (Hou et al., 2023). Similarly, using MS, accurate NMR data, and other complementary computational tools, the structure and absolute stereochemistry of polyol polyketide retinestatin were determined (Huynh et al., 2024). Zhu et al. (2022) isolated huoshanmycins A–C from endophytic *Streptomyces* sp. HS-3-L-1 and determined their structures using 1D/2D NMR and HRESIMS, whereas Shin et al. (2022) used a combination of HRESIMS, 1D/2D NMR, NOESY, ECD calculations, and DP4 + prediction to determine the structures and stereochemistry of streptoglycerides E and H. While software and databases such as SIRIUS and GNPS are useful for metabolite annotations, experimental determination should be preferred. When genetic evidence or heterologous expression is paired with structural characterization, it provides solid evidence linking a molecule to its production process.

### 5.4 Regulatory non-coding RNAs

Post-translational modifications such as phosphorylation, and acetylation as well as two-component systems (TCS) like PhoR-PhoP are fundamental for transferring signals from cells for transcriptional regulation (Martín et al., 2021; Martín and Liras, 2020), and post-transcriptional regulation is controlled by small non-coding RNAs (sRNAs) and non-coding RNAs (ncRNAs). These ncRNAs create intricate regulatory networks that affect both the cell’s development and antibiotic production. Using combined bioinformatics and transcriptomics approaches, hundreds of predicted sRNAs in intergenic regions of *S. clavuligerus* were identified, many of which exhibit stable, highly conserved secondary structures and were shown to directly affect regulatory functions in CA biosynthesis and the direct target genes of the CA BGC for cephamycin C (Caicedo-Montoya et al., 2024).

The complex regulatory pathways of *S. ambofaciens* further confirmed that transcriptomic perturbation led to the discovery of novel small and antisense RNAs that significantly affect primary metabolism, protein synthesis, and spiramycin production. Crucially, these studies suggest that native regulatory RNAs act as manipulable molecular switches (Pinatel et al., 2021). Although a multi-omics approach offers a comprehensive perspective, combining these diverse datasets remains a major computational challenge, resulting in noisy correlations that are difficult to validate biologically. Beyond this, metabolomics platforms without targeted approaches often fail to detect trace or unstable metabolites, so the actual metabolome produced by a cryptic BGC may still be undiscovered.

## 6 Precision toolkits: DNA-level genome editing and pathway mobilization

The application of CRISPR platforms, in combination with synthetic biology, genome mining approaches, and multi-omics techniques, has significantly expanded the range of methods available for natural product discovery and rational strain engineering of *Streptomyces*. Global analysis reveals a hierarchical mechanism for controlling expression at the cluster level. To optimize metabolite production effectively, biosynthetic genes are co-transcribed in operons, whereas regulatory genes are mostly monocistronic. Furthermore, promoter regions in BGCs show enrichment of transcription factor binding sites (TFBSs) with lower binding affinities than those found in core regulon genes, allowing them to readily adapt to environmental signals (Ribeiro Monteiro et al., 2025). Manipulating these native regulatory domains requires precision toolkits to alter transcription, alter toxicity, and refactor entire pathways.

### 6.1 Transcriptional modulation (CRISPRi and CRISPRa)

Beyond simple gene knockouts, CRISPR interference (CRISPRi) enables precise redirection of metabolic flux without permanently altering the DNA sequence. By combining nuclease-deficient Cas9 (dCas9) with repressor domains, researchers can systematically downregulate competing pathways or silence negative regulatory genes. This targeted transcriptional repression has been used to remove metabolic bottlenecks and increase the production of polyketides such as pikromycin, rapamycin, and jadomycin by rerouting cellular resources toward the biosynthetic operons encoding these compounds (Cho et al., 2022; Tian et al., 2020; Ameruoso et al., 2022). A targeted transcriptional repression system has been extremely successful in activating cryptic gene clusters that are toxically knocked out by conventional Cas9 approaches. For example, CRISPRi was used to identify and activate an interesting ~43 kb balmoralmycin BGC from a novel environmental isolate, resulting in the identification of several novel shunt products (Ma et al., 2022). Conversely, CRISPR activation (CRISPRa) employs catalytically inactive Cas proteins (dCas) to activate target genes transcriptionally. It can be used to activate silent biosynthetic pathways or pathway-specific regulators in *Streptomyces* (Zhou et al., 2024). CRISPR-Cas-mediated genome engineering can also activate or enhance natural product biosynthesis by introducing strong promoters or modifying biosynthetic and regulatory pathways, as demonstrated by increased armeniaspirol production in *Streptomyces* sp. A793 (Heng et al., 2023).

### 6.2 Overcoming proteotoxicity and alternative orthologs

High off-target activity and toxicity of CRISPR-Cas9 have been the primary obstacles to genetic editing in *Streptomyces* (Kim et al., 2025). Various strategies have been proposed to address these problems, including Cas9D10A nickase (nCas9)-based genome editing with low toxicity and the application of endogenous CRISPR systems (Ma et al., 2023). In *S. coelicolor* M145, Cas9 can maintain the efficiency of single-gene and gene-cluster deletions while simultaneously reducing cellular toxicity (Ma et al., 2023). In many cases, endogenous type I-E CRISPR systems are favored over heterologously expressed Cas9 because they support enhanced host compatibility, reduced proteotoxicity, and simpler operation, thereby removing the need to deliver exogenous Cas genes (Zhou et al., 2024). Furthermore, the hypercompact Cas12j has been assessed as a replacement editing platform. Interestingly, Cas12j exhibited higher transformation efficiency than SpCas9 and better activation of silent biosynthetic gene clusters (BGCs) than Cas12a in hard-to-manipulate strains such as *Streptomyces* sp. A34053. Although not always globally more efficient than Cas9 or Cas12a, Cas12j provides greater access to previously out-of-reach genomes (Tan et al., 2024).

### 6.3 Advanced precision toolsets: double-strand breaks-free base editing

Traditional CRISPR-Cas9 systems have been developed for more robust genetic manipulations; however, genome instability caused by DNA double-strand breaks (DSBs) and the toxicity of Cas9 remain (Tong et al., 2019). The CRISPR-BEST (CRISPR-Base Editing System) platform was developed, providing both cytidine (CRISPR-cBEST) and adenosine (CRISPR-aBEST) deaminase-based base editors for *Streptomyces* (Tong et al., 2019). CRISPR-BEST enables single-nucleotide-resolution, DSB-free C: G-to-T: A and A: T-to-G: C conversions. Its critical advantage over conventional Cas9 was demonstrated in the non-model actinomycete *S. collinus* Tü365, where CRISPR-BEST successfully introduced premature STOP codons to simultaneously inactivate duplicated kirN genes in the kirromycin biosynthetic pathway, a multiplexed knockout that repeatedly failed with conventional DSB-based Cas9 due to severe genome instability and cellular toxicity (Tong et al., 2019).

Cytosine base editors (CBEs), which combine catalytically impaired Cas proteins with cytidine deaminases, enable DSB-free C•G-to-T•A conversion and can introduce premature stop codons or targeted amino-acid substitutions in *Streptomyces* (Zhao et al., 2020). This approach is particularly advantageous for dismantling competing biosynthetic pathways in industrial chassis. Recently, eSCBE3-NG-Hypa was used to introduce premature stop codons into a competitor pathway gene cluster in *S. avermitilis*, resulting in up to a 4.45-fold increase in avermectin yield compared to the control (Wang et al., 2024). In addition, the CBE base editor dCas9-CDA-ULstr was constructed to introduce internal stop codons in the rapS gene that inhibits rapamycin synthesis in *S. rapamycinicus* 2001, resulting in a 63.3% increase in rapamycin production (Zhao et al., 2020). Furthermore, by incorporating Csy4-based sgRNA processing machinery, CRISPR-BEST supports multiplexed, DSB-free editing from a single plasmid. Further refinements have addressed host-repair limitations of base editing in *Streptomyces*. The uracil intermediate generated by cytidine deamination is typically excised by endogenous uracil DNA glycosylases (UDGs), which lowers editing efficiency. In *S. lividans* 66, permanent UDG inactivation improved editing 3.4–67.4-fold but compromised genome stability, whereas transient UDG disruption by antisense RNA interference (asRNA-BE) increased efficiency 2.8–65.8-fold while preserving genome stability, demonstrating that DSB-free editing in *Streptomyces* can be enhanced by tuning host base-excision repair (Zhang et al., 2021). Such precision toolkits include standard Cas9-mediated gene editing, DSB-free base editing, transcriptional regulation through CRISPRi/CRISPRa, and plasmid-based delivery strategies, as summarized in Figure 2.

**Figure 2:**
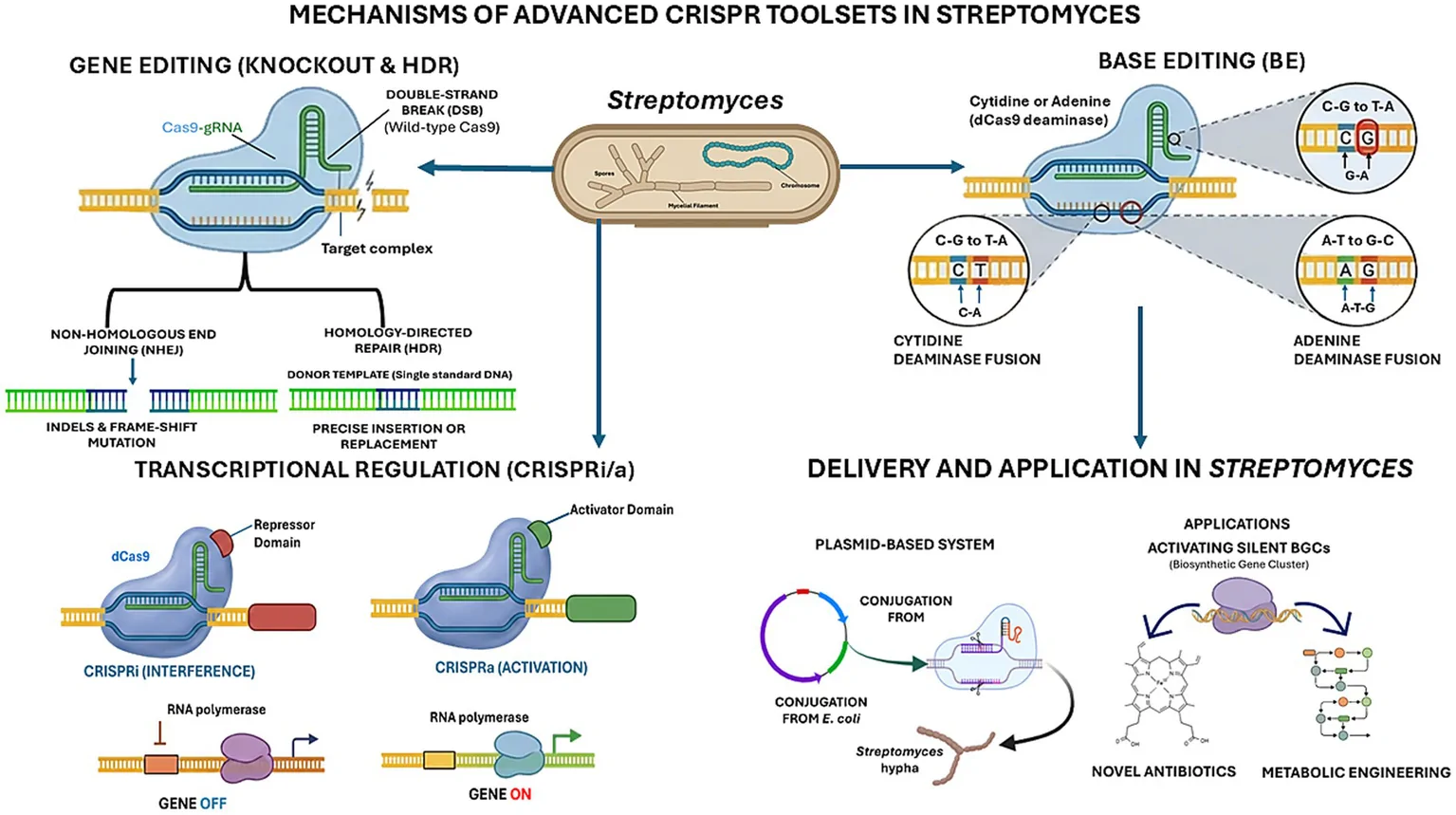
Mechanisms of CRISPR-based toolsets in *Streptomyces*. Upper left: wild-type Cas9 generates double-strand breaks (DSBs) repaired by NHEJ (indels/frameshifts) or HDR with a donor template (precise insertion/replacement). Upper right: DSB-free base editing using Cytidine or adenine deaminase fused to a Cas-derived DNA-targeting protein (C•G-to-T•A and A•T-to-G•C). Lower left: transcriptional regulation by CRISPRi (repressor domain, gene OFF) and CRISPRa (activator domain, gene ON). Lower right: plasmid delivery by conjugation from *E. coli* and applications in silent BGC activation, novel antibiotic discovery, and metabolic engineering.

### 6.4 High-throughput one-pot refactoring platforms

The Direct Pathway Synthesis and Editing (DiPaSE) platform is a next-generation synthetic biology strategy for rapid refactoring of high-GC *Streptomyces* biosynthetic gene clusters (BGCs). Activating cryptic BGCs requires coordinated use of environmental signals, regulatory rewiring, and genome-engineering strategies. This approach identified previously uncharacterized genes in the aureothin BGC, and NP concentrations increased in the native host, offering a direct route from *in silico* database prediction to functional, high-yielding polyketides (Takeda et al., 2026). These precision toolkits offer molecular scissors and controls to edit the genome, but activating cryptic BGCs requires coordinated use of environmental signals, regulatory rewiring, and genome-engineering strategies.

### 6.5 *In vivo* pathway mobilization in the native *Streptomyces* host

Shifting from minor regulatory adjustments to large-scale pathway manipulation requires genome-mobilization strategies. ACTIMOT (Advanced Cas9-mediaTed *In vivo* Mobilization and multiplication of BGCs) artificially mimics the spread of antibiotic-resistance genes: a release plasmid (pRel) carrying a *Streptomyces* replicon and CRISPR-Cas9 excises the chromosomal target DNA region, and a capture plasmid (pCap) carrying a multicopy replicon relocates and multiplies it, enhancing BGC expression in a gene-dosage-dependent manner without the intermediate *E. coli* host required by classical cloning (Xie et al., 2024; Bian, 2025). Applied to *S. coelicolor* M145, *S. avidinii* DSM40526, and *S. armeniacus* DSM19369, ACTIMOT mobilized target regions of up to 149 kb and unlocked 39 previously unexploited compounds across four classes, avidistatins, avidilipopeptins, mobilipeptins, and actimotins, in native species or the heterologous host *S. albus* Del14 (Xie et al., 2024).

### 6.6 *Ex vivo* multiplexed refactoring of multioperon BGCs (yeast and *E. coli* platforms)

A major challenge in activating cryptic BGCs is that they often comprise multiple operons requiring simultaneous replacement of several native promoters. Because direct multiplexed editing of the native *Streptomyces* chromosome remains difficult, several platforms reconstruct the cluster ex vivo and then transfer the refactored BGC to an expression host. The mpCRISTAR system (Multiple Plasmids-based CRISPR/Cas9 and TAR) overcomes the limitations of single-array CRISPR systems by co-transforming multiple gRNA-expressing plasmids, each carrying a unique auxotrophic marker, into *S. cerevisiae*; using TAR, up to four promoters of a model cluster were replaced with nearly 100% efficiency, multiplexing was expanded to six (68%) and eight (32%) promoters, and iterative marker recycling enabled refactoring regardless of operon complexity (Kim, et al., 2020). The CRISETR system combines CRISPR/Cas9 with RecET-mediated recombineering in *E. coli*; applied to the 74-kb daptomycin BGC, it reconstructed multiple promoter combinations, yielding a 20.4-fold increase in daptomycin production after transfer to the production host (He et al., 2024). Likewise, iCASRED is a scarless *E. coli* editing platform coupling site-specific serine integrases with Cas9-mediated recombination for simultaneous integration, deletion, or activation of multiple BGCs before heterologous transfer (Zheng et al., 2025).

## 7 Activation triggers: ecological, nucleoid-level, and transcriptional switches

### 7.1 Environmental activation and One Strain Many Compounds strategies

Low-intervention methods such as the OSMAC framework rely on cultivating microorganisms under various growth conditions, variable carbon sources, trace metals, and aeration to activate cryptic BGCs. For example, simultaneously changing carbon sources and inhibiting butenolide signaling has been shown to synergistically activate silent clusters and improve polyketide production by more than 50-fold (Li et al., 2022). Altering nutrient profiles and microbial co-cultivation stimulates ecological competition, which acts as a potent chromatin modifier. Co-culturing *Streptomyces* with coevolving bacteria (e.g., *Rhodococcus* or *Pandoraea* species) often induces the production of novel aromatic glycosides and pigments that remain completely silent in monoculture, indicating how ecological signaling can be seamlessly combined with genome mining to unlock chemical diversity (Guerrero Garzón et al., 2025; Huynh et al., 2021).

### 7.2 Nucleoid-level and chromatin-level regulation of BGCs

Beyond transcription factors and promoter engineering, the nucleoid-level landscape and physical chromatin topology of *Streptomyces* are key determinants of BGC silencing yet remain largely underexplored. In actinomycetes, the nucleoid-associated protein (NAP) Lsr2 (a functional equivalent of H-NS in *Escherichia coli*) primarily binds AT-rich regions of the genome. It oligomerizes to form a higher-order repressive nucleoprotein complex that physically traps RNA polymerase or blocks its binding, silencing cryptic BGCs, many of which are acquired by horizontal gene transfer (Zhang et al., 2021). While functionally analogous to eukaryotic heterochromatin in its silencing outcome, this mechanism is distinct from histone-based repression. Removing the lsr2 gene serves as a strong global nucleoid-level switch that, by eliminating this topological repression, enables the production of previously silent metabolites such as warkmycins (Cuervo et al., 2024). In addition to nucleoid-associated proteins, secondary messengers can remodel the metabolic landscape to activate cryptic pathways. For example, overexpressing the diguanylate cyclase gene cdgB to increase intracellular c-di-GMP levels activates silent type II PKS pathways, enabling the identification of novel tricyclic aromatic polyketides with cytotoxic properties (Zhang et al., 2025). This nucleoid-level regulation is further fine-tuned by a complex interplay of NAPs and pathway-specific transcription factors that control RNA polymerase’s access to DNA, as illustrated by the pathway-specific regulator CmlR, which counteracts silencing in *S. venezuelae* (Zhang et al., 2021).

Recognizing the power of these topological and nucleoid-level barriers, synthetic biologists are developing less destructive ways to bypass them. For example, the small type V-F nuclease AsCas12f1 has been developed as a low-toxicity, high-efficiency genome editing tool for streptomycetes: owing to its small size and weak DNA cleavage activity, it achieved higher transformation efficiencies than SpCas9 or FnCpf1, 100% efficiency for single-gene and gene-cluster deletions in *S. coelicolor*, multiplexed two-gene (46.7%) and two-cluster (40%) deletions, and efficient editing in the recalcitrant *S. hygroscopicus* SIPI-054, where SpCas9/FnCpf1 perform poorly (Hua et al., 2024). Concisely, low-toxicity nucleases present a feasible substitute for large SpCas9 when regulatory regions are difficult to manipulate. As a future direction, fusing catalytically dead Cas proteins to activation domains or nucleoid-level modifiers could enable site-directed BGC activation without permanent DNA-sequence changes.

### 7.3 Regulatory rewiring and internal Signaling circuits

Environmental stimuli provide the initial trigger; sustained BGC activation often requires the dismantling of native regulatory checkpoints and the precise balancing of metabolic flux. Global and pathway-specific transcription factors are the main bottlenecks for cryptic BGC expression. Deleting global repressors, for example, the wblA gene in *S. ghanaensis* (Makitrynskyy et al. 2025), or specific repressors such as the MarR-family regulator in *S. formicae* (Devine et al., 2021), results in reprogramming of native barriers to the upregulation of multiple biosynthetic transcripts and significant increases in compounds. Similarly, the overproduction of pathway-specific activators, like the MerR-family transcription factor in *S. vilmorinianum* (Zhao et al., 2025), can increase precursor supply and product formation of compounds like granaticin B. Introducing new signaling pathways, for instance, the recognition of γ-butenolide-type molecules (e.g., SFB1) that activate specific receptors like TylP in *S. fradiae*, enables further control of the internal mechanisms (Chen et al., 2025).

### 7.4 High-expression promoter libraries and decoy systems

Completely changing the complex native regulations, synthetic promoter libraries and transcription factor decoys offer tunable regulation. Strongly expressed or stress-inducible promoters, such as the KCl-stimulated kasOp* promoter in *S. albus,* can lead to the production of hidden metabolites like coprisamides and padanamides (Wang et al., 2025). Beyond single promoter insertions, systematic promoter rebalancing can change the profile of secondary metabolites. For instance, randomized ermEp1 promoter libraries have been successfully applied to the pamamycin BGC, reprogramming its biosynthetic flux toward rare, higher molecular weight derivatives, including the highly potent anticancer compound pamamycin 663A (Eckert et al., 2023). Concurrently, transcription factor (TF) decoy plasmids with synthetic binding sites can capture the negative regulators; for example, multicopy decoys for *S. avermitilis* AfsR and AveI repressors disable their inhibitory effect, boosting avermectin production over fivefold (Huang et al., 2025). To attain precise, tunable control, synthetic promoter libraries (SPLs) have been rationally designed with different degrees of spacer randomization and conserved ribosome-binding sites to prevent cross-recombination while offering a comprehensive dynamic range of expression (Shil et al., 2026).

Activating a silent BGC also requires sufficient metabolic flux to support the substantial biosynthetic burden. Knocking out competing pathways or redirecting primary metabolism can significantly improve precursor supply. For instance, in *S. roseosporus*, CRISPRi-mediated repression of aspartate-competing genes (acsA4, pta, pyrB, pyrC) combined with the deletion of a competing red-pigment BGC significantly redirects metabolic flux, resulting in a 2.6-fold increase in daptomycin titers (Li et al., 2024). Similarly, overexpressing the tetR regulator XNR_0706 in *S. albus* activates fatty acid β-oxidation, enhances precursor supply, and increases heterologous spinosad production (Cui et al., 2025). Avoiding early precursor exhaustion, dynamic control systems such as quorum-sensing-mediated EQCi regulate multiple targets simultaneously and regulate the balance between primary and secondary metabolism without an observed growth penalty (Tian et al., 2020). Furthermore, genome-scale metabolic models (GEMs) combined with flux scanning, such as the Salb-GEM (Kittikunapong et al., 2021), or dynamic triacylglycerol degradation (ddTAG) systems (Wang et al., 2020), provide predictive and temporal control over precursor availability, which, in turn, leads to industrial-level production of compounds such as avermectin B1. However, genetic rewiring and metabolic balancing can impose genetic instability over successive generations, resulting in ‘production strain degeneration’ upon prolonged industrial fermentation. Besides, low-throughput empirical approaches like OSMAC are barely scalable. This instability and the disadvantages of entirely transcriptional controls highlight the necessity of considering the physical arrangement of the genome itself instead of only the transcription factors.

### 7.5 Artificial intelligence (AI)-driven promoter design and metabolic flux modeling

Computational platforms provide powerful means for uncovering the genetic potential of *Streptomyces*; however, fully harnessing this potential is hampered by a major limitation: the lack of standardized, well-characterized genetic parts. In contrast, in model organisms such as *Escherichia coli* (Wang et al., 2020) and *Saccharomyces cerevisiae* (Vaishnav et al., 2022), deep learning approaches have already been successfully employed to generate entirely novel promoter sequences with a high fraction of functional constructs. *Streptomyces*, by comparison, have historically posed difficulties due to their complex, GC-rich genomes and their diverse sigma-factor regulatory networks. Very recently, deep generative modeling has begun to address this gap. Zhou et al. (2026) computationally designed approximately one billion synthetic *Streptomyces* promoters using a Wasserstein generative adversarial network with gradient penalty (WGAN-GP) trained on cross-species dRNA-seq and RNA-seq datasets. Among the experimentally tested candidates, 92% were functional, and the measured promoter activities spanned a remarkably broad dynamic range of 17,100% (0.08–17.10-fold relative to ermE*p*), with seven variants outperforming the strong kasOp benchmark. Notably, these synthetic promoters exhibited very low sequence similarity to the native *Streptomyces* genome, retained robust activity across four phylogenetically distinct *Streptomyces* hosts, and increased compounds of polycyclic tetramate macrolactams 28.6-fold, daptomycin 25.7-fold, and rapamycin 6.1-fold, providing the sequence orthogonality needed to minimize host crosstalk and support independent gene expression (Zhou et al., 2026).

For instance, the Salb-GEM genome-scale metabolic model for *S. albus* J1074 (Kittikunapong et al., 2021) exemplifies how GEMs (genome-scale metabolic models) have been useful for predicting *in silico* metabolic flux patterns and for identifying bottleneck precursors, such as malonyl-CoA depletion. Although AI-optimized promoters and genome-scale metabolic modeling have each achieved major success on their own, an automated “holistic system assembly” platform remains an aspirational goal rather than a demonstrated reality. Future efforts to couple AI-generated, sequence-orthogonal promoter libraries with biology-based modeling hold great promise. Ultimately, the whole system will help researchers not only predict but also rationally design promoters that can balance metabolism without increasing proteotoxic stress, enabling them to move beyond empirical trial and error.

## 8 Reprogramming the polyketide assembly line and host chassis

### 8.1 CRISPR-based engineering of polyketide catalytic machinery

Modern synthetic biology extends beyond transcriptional regulation, with efforts to directly reprogram the catalytic machinery of the polyketide assembly line. CRISPR/Cas9 systems facilitate the precise deletion, replacement, or domain-swapping of modular PKS genes, enabling the redirection of metabolic flux toward high-yielding or novel scaffolds. For example, targeted substitutions of specific PKS modules with heterologous complexes have successfully redirected biosynthetic pathways to produce hybrid polyketides such as milbemycin D as an industrial compound (Tao et al., 2026). Moreover, the targeted insertion of strong constitutive promoters directly upstream of cryptic PKS operons effectively bypasses native cellular checkpoints and has led to the biosynthesis of novel polyketide scaffolds such as venediol without whole-pathway refactoring (Li et al., 2022), while precise module-swapping has been used to integrate the venediol PKS into the pikromycin assembly line to generate novel hybrid macrolides (Wang et al., 2025).

### 8.2 Characterization of hybrid and novel polyketide scaffolds

Synthetic biology techniques have led to the development of hybrid polyketide pathways enabling both the discovery of novel structural motifs and the elucidation of complex tailoring enzymology. Targeted domain swapping and reverse genetics allow researchers to systematically determine the catalytic necessities of hybrid assembly lines. For example, targeted mutagenesis of tailoring domains, such as NAD(P)H-dependent ketoreductases, has greatly helped to verify the biosynthetic domain of phenazine-polyketide hybrids like nexphenazine A, to identify key branch-point shunt metabolites (Wang et al., 2020), and to confirm modular PKS functions via base editing (Otsuka et al., 2025). Instead of focusing on individual pathways, the engineered multiplexed chassis strains enable simultaneous activation of multiple biosynthetic pathways. The stepwise incorporation of several heterologous pathways into the efficient hosts resulted in the generation of libraries of various core structures, including benzoisochromanequinones, angucyclines, and tetracenomycins—thereby proving the power of chassis engineering to act as a universal platform for structural diversification (Zhu et al., 2021; Zou et al., 2025).

Exploiting natural pathway crosstalk can yield unprecedented hybrid scaffolds. In *S. pactum*, the interaction between NFAT-133 and conglobatin PKS pathways results in hybrid compounds (Zhou et al., 2022), while the discovery of hybrid Type I/Type III PKS systems (e.g., resorculins) and PKS-NRPS cassettes (e.g., colibrimycins) highlights the modular flexibility of these assembly lines when native regulatory limits are bypassed (Lacey et al., 2023; Prado-Alonso et al., 2022). Ultimately, these strategies extend beyond pharmaceuticals, as demonstrated by the discovery of anti-aphid polyketides like strekingmycins, highlighting the utility of genome mining for next-generation biopesticides (Wang et al., 2023). The above information suggests that targeted modification of PKS domains, regulatory limits, and intermediate flux can enable large-scale chemical diversification. Thus, producing hybrid polyketides evolves from a spontaneous side effect of pathway interference into a strategic outcome in synthetic biology (Table 2). The major synthetic biology strategies enabling this rational redesign and structural diversification are illustrated in Figure 3.

**Table 2:** Synthetic biology approaches to produce novel and hybrid polyketide-type secondary metabolites in *Streptomyces* species.

Synthetic biology approaches	Process	*Streptomyces* used and studied	Used method	Remarks	Ref
Competitive pathway removal and precursor supply	CRISPRi of Asp-competing genes (acsA4, pta, pyrB, pyrC); deletion of red-pigment BGC; kasOp* replacement of dptEp; duplication of engineered dpt* cluster	*S. roseosporus* NRRL 11379 (case study)	CRISPRi + homologous-recombination promoter replacement/BGC deletion	Daptomycin 350.7 μg/mL (2.6 × WT); highest shake-flask titer reported	Li et al. (2024)
Promoter Engineering	kasOp* insertion upstream of vedA (after pikAI deletion)	*S. venezuelae* ATCC 15439 (case study) (*S. coelicolor* M1152) (standard chassis)	CRISPR-Cas9	Activated cryptic type I PKS; venediols A/B; non-collinear modular PKS	Li et al. (2022)
PKS Domain Modification	*npzI inactivation (NAD(P)H-dependent ketoreductase)*	*Streptomyces* sp. KIB-H483 (case study)	Gene inactivation (λ-Red) + *in vitro* enzymology	Abolished nexphenazine A; released stereoselectively tailored shunt intermediates	Wang et al. (2022)
Regulatory Rewiring	In-cluster TCS (MerR-family Orf10/Orf11) overexpression	*S. vilmorinianum* YP1 (case study)	Overexpression	A record granaticin B titer 716.27 mg/L (3.0-fold; 4.36-fold granaticin A)	Zhao et al. (2025)
Dynamic Balancing	EQCi system (QS-CRISPRi)	*S. rapamycinicus* (case study)	Synthetic circuit	~660% higher rapamycin (1836 mg/L) without growth penalty	Tian et al. (2020)
Dynamic Balancing and Gene Dosage	Multi-copy BGC integration + QS-responsive CRISPRi (*sbbAp*) targeting primary metabolism (*gltA*, *fabH*)	*S. hygroscopicus* SIPI-054 (case study)	CRISPRi + Site-specific recombination	3.4-fold increase to 6.14 g/L milbemycin A3/A4; autonomous flux balancing prevents growth arrest	Xu et al. (2026)
Base Editing	Target-AID editing of *ladA5* PKS	*S. lavendulae* FRI-5 (case study)	Base Editing	Complete loss of lavencidin confirmed modular PKS function	Otsuka et al. (2025)
Pathway Crosstalk Optimization	Sequential in-frame inactivation of competing pathways (nftCD, congC, ptmA) and pieA5 (piericidin PKS module 6)	*S. pactum* ATCC 27456 (case study)	In-frame gene deletion (homologous recombination)	Prematurely offloaded pentaketide intermediates reveal PKS offloading and promiscuous tailoring enzymes	Alharbi et al. (2025)
Lipid Metabolism	TetR regulator XNR_0706 overexpression + 0.3 g/L fatty-acid (linoleic/oleic) supplementation	*S. albus* B4 (heterologous spinosad chassis) (standard chassis)	TF overexpression	Activates fatty-acid β-oxidation; 20.6% higher spinosad, +42.4% further with fatty-acid feeding	Cui et al. (2025)

**Figure 3:**
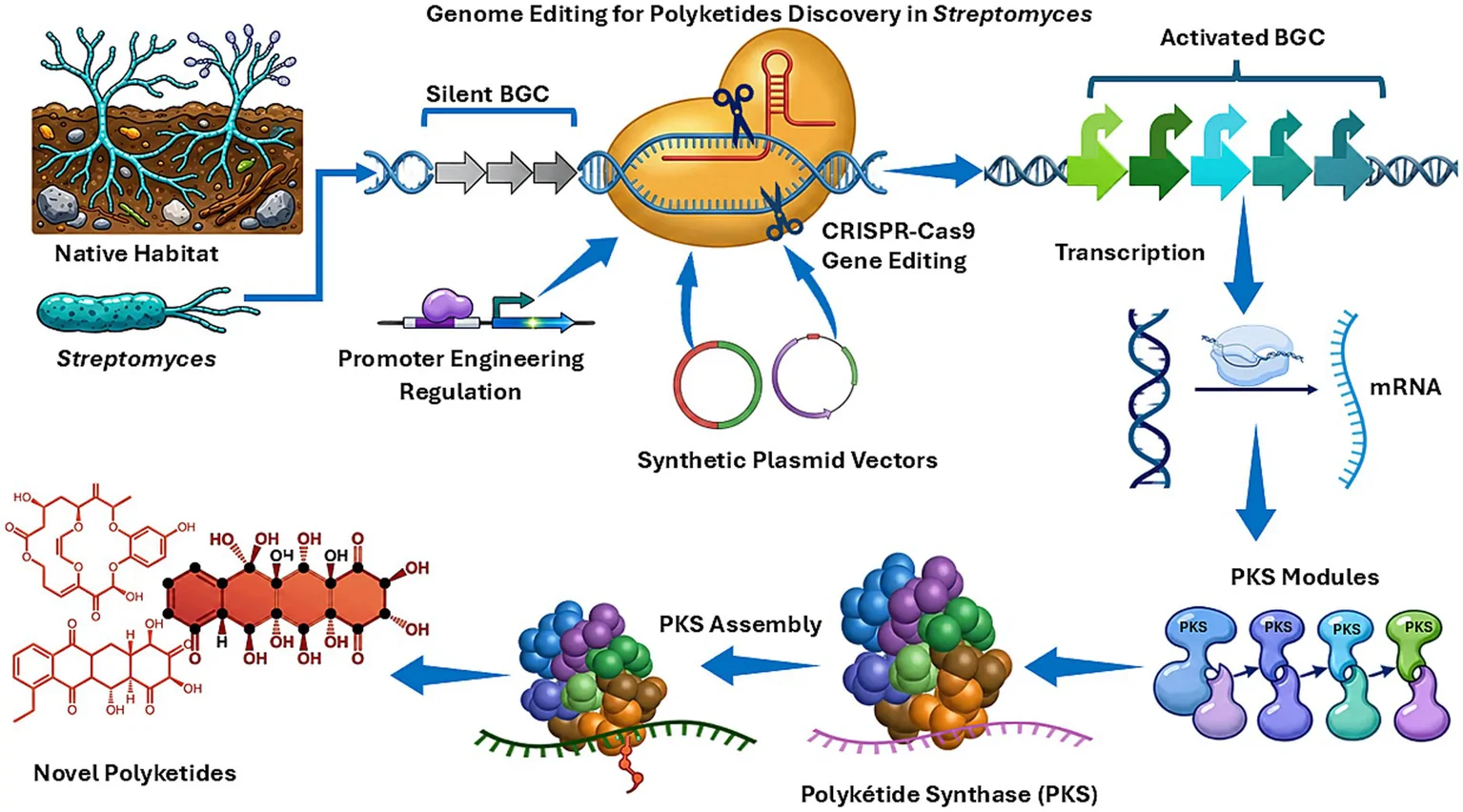
Genome-editing and promoter-engineering strategies for polyketide discovery in *Streptomyces*. Silent BGCs from native isolates are activated by CRISPR-Cas9 editing, promoter engineering, and synthetic plasmid vectors; activated BGCs are transcribed and translated into PKS modules whose assembly-line activity yields novel polyketide scaffolds.

### 8.3 Heterologous expression platforms and chassis optimization

The success of heterologous BGC expression depends strongly on the regulatory compatibility, precursor availability, and metabolic state of the host. Although model hosts offer reliable platforms, genome-reduced and chassis-optimized strains are achieving better results by minimizing precursor competition (Table 3). For instance, by deleting multiple endogenous BGCs and developing an efficient chassis (*S. albus* ZD12), the metabolic burden and background chemical noise are both drastically reduced, resulting in multi-fold increases in heterologous spectinabilin yields compared to traditional hosts (Wang et al., 2026). The removal of endogenous type II polyketide clusters in industrial strains such as *S. aureofaciens* ‘Chassis2.0’ was reported to increase native oxytetracycline titers by 370% and to enable the discovery of new pentacyclic polyketides (Zou et al., 2025). Identification of the pqq gene cluster as a universal PKS enhancer factor that elevates ATP and NADPH pools has led to up to a 40-fold increase in production of various polyketides on multiple *Streptomyces* host chassis (Wang et al., 2024). Combining BGC refactoring and Genome-scale metabolic model (GEM) prediction engineered an efficient heterologous pathway for the overproduction of oviedomycin, resulting in production up to 670 mg/L in *S. coelicolor* (Gu et al., 2023). Innovative translational rescue strategies have also been successfully employed to alleviate the bottlenecks for efficient translation of large-sized PKS mRNA molecules by fragmenting them into differently translatable subunits via incorporation of heterologous docking domains, which dramatically enhanced the production of butenyl-spinosyns and avermectins (Liu et al., 2025). These genome-minimized platforms can be a game-changing approach to manufacturing bioproducts at scale and tapping into silent chemical diversity (Xu et al., 2025). These findings illustrate the potential of genome-minimized industrial chassis for large-scale biomanufacturing (Figure 4).

**Table 3:** *Streptomyces* host platforms for heterologous BGC expression. Genome-reduced chassis strains are engineered by deletion of endogenous BGCs, and standard heterologous expression hosts are used without genome reduction.

Category	Host strain	Engineering strategy	Expressed BGC/metabolite	Key advantage/effect	Ref
Genome-reduced *Streptomyces* chassis strains	*S. albus* ZD12	Deletion of 4 endogenous BGCs	Spectinabilin	2.6-fold higher yield than J1074; minimized precursor competition	Wang et al. (2026)
	*Streptomyces* sp. A4420 CH	Deletion of 9 native polyketide BGCs	4 distinct heterologous polyketide BGCs	Polyketide-focused chassis; outperformed classical hosts (M1152, TK24, J1074) across all tested conditions; robust growth and sporulation	Klumbyš et al. (2024)
	*S. albus* J1074	Heterologous expression of marine-derived type II PKS cluster	8 angucyclines incl. 4 novel	Diversified angucycline scaffolds; cytotoxic activity	Zhang et al. (2024)
Heterologous expression hosts (non-genome-reduced)	*S. lividans* ΔYA6/TK24	Standard heterologous expression hosts	Pentangumycin, SEK90, Marinomycin, Bisenarsan	Versatile PKS expression; targeted genome mining	Gummerlich et al. (2020); Abraham et al. (2024); Hoshino et al. (2025)
	*S. albus* J1074	Heterologous expression of *Kutzneria* sp. CA-103260 BGC	Epemicins A and B (30-membered macrolides)	Expression from a phylogenetically distant actinomycete genus	Kontou et al. (2021)
	*S. albus* J1074	Heterologous expression of metagenome-derived type II PKS cluster	Metathramycin (aureolic acid polyketide)	Activation of BGC from uncultured environmental DNA	Stevenson et al. (2021)
	*S. coelicolor ∆ppk*	*ppk* deletion (polyphosphate kinase knockout); integration of *elm* cluster from *S. olivaceus* Tü2353	8-Demethyl-tetracenomycin C (8-DMTC; aromatic polyketide)	Actinorhodin-overproducer background; 8-DMTC production caused mycelial fragmentation and abolished sporulation	Cinar et al. (2021)
Native production	*S. aureofaciens*	Deletion of 2 endogenous Type II PKS clusters	Oxytetracycline, TLN-1	370% increase in titers; enabled novel penta-ring polyketide discovery	Zou et al. (2025)

**Figure 4:**
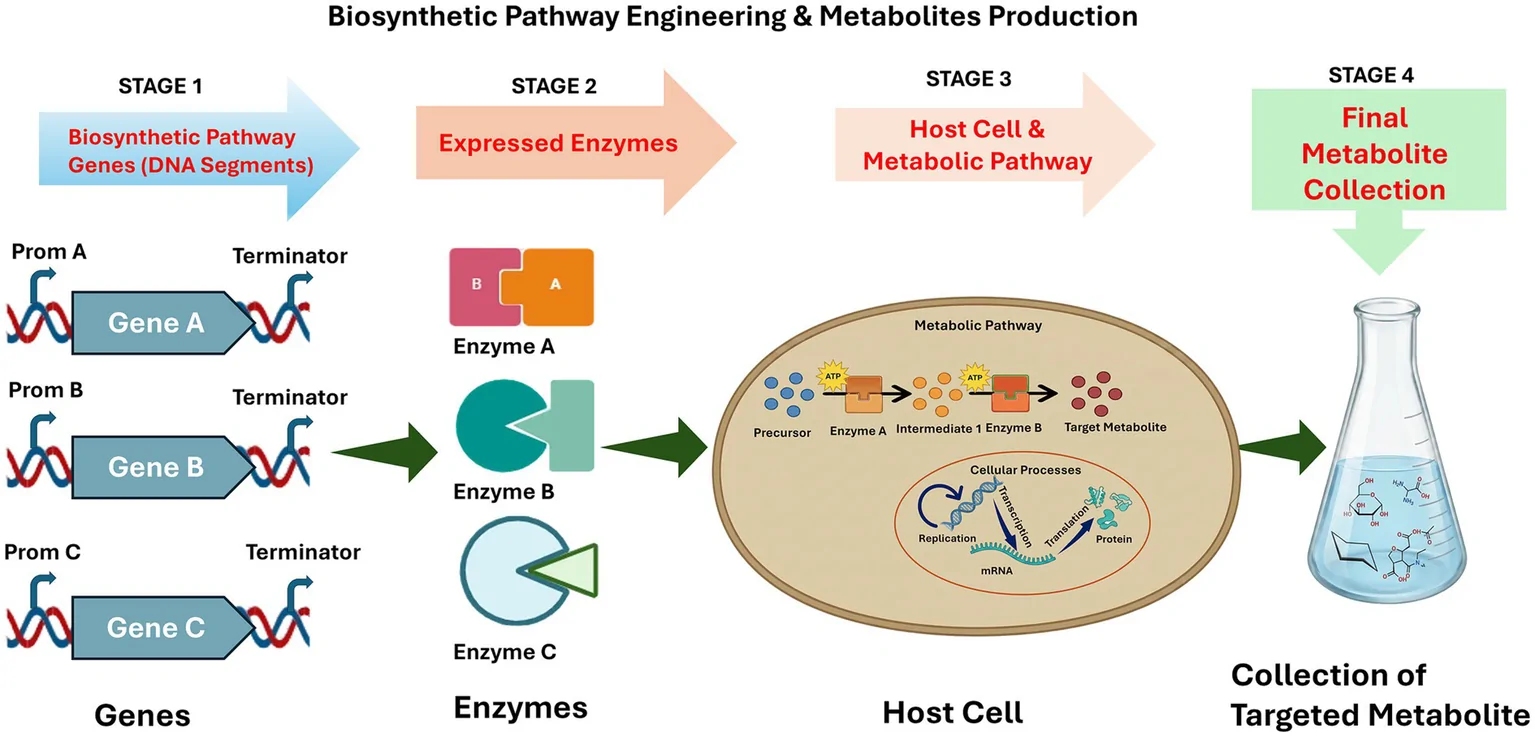
Conceptual workflow of biosynthetic pathway engineering in a heterologous host. Stage 1: assembly of biosynthetic genes with promoters and terminators. Stage 2: expression of the corresponding enzymes. Stage 3: reconstitution of the metabolic pathway in the host cell, converting precursors through intermediates to the target metabolite. Stage 4: collection of the target metabolite.

The *S. albus* J1074 strain demonstrates remarkable versatility for expression of BGCs from various phylogenetically distant actinomycete genera. The production of the 30-membered macrolide epemicins, normally isolated from *Kutzneria* sp. C A − 103,260, was successfully achieved via the heterologous expression of the biosynthetic gene cluster in *S. albus* J1074, enabling genetic manipulation of the rare actinomycete pathway (Kontou et al., 2021). Another example is metathramycin, a new aureolic acid compound isolated from environmental DNA (metagenome), which confirms the ability of *S. albus* J1074 as a universal host for activation of BGCs with uncultured organisms (Stevenson et al., 2021). Besides that, metabolic mutants are also used as heterologous expression hosts. The actinorhodin-overproducer *S. coelicolor* ppk mutant, lacking polyphosphate kinase, was tried as a scaffold for expressing the elm gene cluster of *S. olivaceus* T2353. Although obtaining 8-demethyl-tetracenomycin C (8-DMTC) production was a success, production in recombinant strains was accompanied by a severe disturbance of morphology, such as mycelial fragmentation and loss of sporulation, showing that morphology-compatible selection of a host organism might be required for efficient polyketide expression in heterologous systems (Cinar et al., 2021).

Beyond classical model hosts, phylogeny-guided chassis development has yielded specialized platforms such as *Streptomyces* sp. A4420 CH, engineered by deleting nine native polyketide BGCs. This polyketide-focused chassis maintained robust growth and sporulation while successfully expressing four distinct heterologous polyketide BGCs under all tested conditions, consistently outperforming established hosts including *S. coelicolor* M1152, *S. lividans* TK24, and *S. albus* J1074 (Klumbyš et al., 2024). This highlights the value of expanding the host panel beyond traditional models for polyketide discovery.

## 9 Challenges for industrial translation

Genetic optimization can enhance metabolic effectiveness and production potential of *Streptomyces*; successful industrial translation depends on strain stability, fermentation performance, morphology, and oxygen transfer. Current advances in *Streptomyces* chassis have adopted a process-oriented approach to ensure stable performance in large-scale fermentation (Chen et al., 2026).

*Metabolic burden and stability:* Overexpression of biosynthetic genes using strong constitutive promoters (e.g., *kasOp*, *hrdBp*) imposes a significant metabolic burden by increasing ATP, NADPH, and precursor pools, leading to slower growth and decreased yields (Wang et al., 2013). Promoter engineering and stage-specific expression, including SP-series and sf14p variants, maintain precursor supply and enhance production, lowering metabolic stress in the cell (Mao et al., 2024). In *Streptomyces*, primary metabolic flux and energy-containing compounds (e.g., NADPH and ATP) affect the transition from vegetative growth to secondary metabolite biosynthesis (Wang et al., 2020; Krysenko and Wohlleben, 2024). Dynamic or feedback-controlled promoters, responsive to pathway intermediates or quorum-sensing signals, adjust metabolic burden by temporally maintaining gene expression with the cell’s physiological state (Mao et al., 2024; Xu et al., 2022). Likewise, production strains should maintain genetic and phenotypic stability throughout the cultivation process and multiple fermentation cycles.

*Pellet morphology and oxygen transfer:* Pellet morphology and oxygen transfer influence the metabolite production of *Streptomyces*. For example, talc microparticles addition to *S. rimosus* shake-flasks altered pellet size depending on the initial nitrogen concentration and increased oxytetracycline, ADOTC, and spinoxazine A up to 4-, 5-, and 1.6-fold (Ścigaczewska et al., 2024), in 5 L *S. rimosus* bioreactors, the same microparticles increased rimocidin 17-fold the bioreactors inoculated with spores, but conferred no benefit, and in some cases a negative effect, when inoculated with a preculture (Ścigaczewska et al., 2024), and in *S. clavuligerus*, baffled (i.e., high-shear) flasks gave smaller and faster-disintegrating pellets as well as higher clavulanic acid than in low-shear geometries which were probably relieved from oxygen limitation at the pellet core (Yepes-García et al., 2020). These results reveal that the correlation between morphology (structure) and productivity is determined by strain background, inoculum, nutrient conditions, and bioreactor hydrodynamics, not only by the size of the pellets.

*Titer, productivity, and scale-up:* Industrial performance assessed using both final compounds and production volumes, along with fermentation period and process potential. Fed-batch methods maintain substrate availability and control key fermentation parameters during the production stage. For example, dramatic supplementation of isopropanol, alanine, pyruvate, and nicotinamide, followed by glucose feeding, increased amphotericin B production to 18.39 g/L in a 5-L fermentor, indicating an 85.9% enrichment over batch fermentation (9.89 g/L). This example highlights how combining metabolic precursor supplementation with controlled resource methods can increase polyene macrolide production (Zhang et al., 2020). For instance, the *Streptomyces* multiplexed artificial control system (SMARTS) can achieve this consistently and reliably in 120 m^3^ industrial fermenters, yielding 8.4 g/L baiweimycin (Yang et al., 2025). Further, systems-level engineering, particularly the 5 M model (e.g., dynamic degradation of intracellular triacylglycerols), increased avermectin B1a by 50%, reaching 9.31 g/L in a 180 m^3^ industrial fermentation (Wang et al., 2020). Besides, the StSS method showed good scalability: a 4 L fermentor batch produced undecylprodigiosin (a polyketide prodiginine) at 1.1 g/L after only 4 days with a productivity rate of 9.17 mg/L/h, the highest amount published till then for this compound, which illustrates how systems that combine primary and secondary metabolism stay both stable and productive beyond the shake-flask scale level (Zhao et al., 2022). These examples provide evidence that chassis-level genetic engineering could support large-scale metabolite production.

An integrated, multifaceted metabolic engineering structure that combines competitive pathway elimination, gene dosage amplification, and dynamic CRISPRi regulation was developed to overproduce milbemycin in *S. hygroscopicus* SIPI-054. Knockout of three competing BGCs resulted in a 34.2% increase in milbemycin A3/A4. Integrating one or three extra BGC copies via site-specific recombination further enhanced compound production by 71.1–112.8%, and a CRISPRi circuit that responded to quorum sensing and targeted citrate synthase (gltA) and β-ketoacyl-ACP synthase (fabH) led to a redistribution of carbon flux without growth arrest. By combining these metabolic engineering strategies, the strain was engineered optimally and reached the highest shake-flask titer of milbemycin A3/A4 reported as 6.14 g/L, representing a 3.4-fold increase over the original strain (Xu et al., 2026).

## 10 Concluding remarks and future perspectives

Over the past decades, advances in synthetic biology strategies have transformed natural product discovery in the genus *Streptomyces*. These approaches enable the activation of cryptic or poorly expressed BGCs into highly accessible platforms for industrial compound discovery and production (Nguyen et al., 2020). The integration of multi-omics remains at the heart of this discovery pipeline, integrating deep genomic mining, transcriptomic signatures, and high-resolution untargeted metabolomics to link previously uncharacterized genotypes to newly observed polyketide phenotypes. With the support of multi-omics approaches, synbio-enabled CRISPR-Cas tools across the Cas9, Cas12, and hypercompact Cas12j platforms improve the manipulation of complex, high-GC-content *Streptomyces* genomes, enabling the systematic, orchestrated refactoring of highly complex PKS pathways.

A consistent set of engineering tools, including heterologous expression in genome-minimized chassis strains, integrase-assisted genome editing (e.g., iCASRED), and combinatorial domain swapping, has demonstrated significant potential in addressing long-standing bottlenecks in polyketide discovery. Furthermore, a major step forward has been achieved with advanced DSB-free base-editing technologies (such as cytidine and adenine base editors, e.g., CRISPR-BEST and eSCBE3-NG-Hypa). These tools allow the introduction of premature stop codons or targeted point mutations within competitive metabolic pathways without causing lethal double-strand breaks, facilitating the activation and characterization of cryptic polyketides in their native or heterologous hosts.

In the future, multiplexed, genetically co-optimized *Streptomyces* chassis strains are expected not only to consolidate current conceptual and computational frameworks but also to enable a new paradigm of such integration, which remains in early proof-of-concept stages. Although AI-designed promoters have already shown substantial success (Zhou et al., 2026), a critical next step is to train these models specifically on GC-rich *Streptomyces* promoter regions, as most existing generative architectures exhibit a pronounced bias toward *Escherichia coli*–derived training datasets. Furthermore, future studies on AI-assisted strain design and process optimization may eventually contribute to more predictable fermentation outcomes. However, integration of these components remains at an early proof-of-concept stage.

## Footnotes

1
https://webofknowledge.com

2
https://scholar.google.com

3
https://www.scopus.com/home.uri
